# A novel de novo GABRA2 gene missense variant causing developmental epileptic encephalopathy in a Chinese patient

**DOI:** 10.1002/acn3.52262

**Published:** 2024-12-31

**Authors:** Li Yang, Xingyu Wan, Ran Hua, Junhong Jiang, Baotian Wang, Rui Tao, De Wu

**Affiliations:** ^1^ Department of Pediatrics the First Affiliated Hospital of Anhui Medical University Hefei Anhui P. R. China; ^2^ Department of Pediatrics The People's Hospital of Hanshan County Hanshan Anhui P. R. China; ^3^ Second School of Clinical Medicine Anhui Medical University Hefei Anhui P. R. China; ^4^ Department of Psychiatry Chaohu Hospital of Anhui Medical University Hefei China; ^5^ Department of Psychiatry, School of Mental Health and Psychological Sciences Anhui Medical University Hefei China; ^6^ Department of Psychiatry Anhui Psychiatric Center Hefei China

## Abstract

**Background:**

Variants in the *GABRA2* gene, which encodes the α2 subunit of the γ‐aminobutyric acid A receptor, have been linked to a rare form of developmental and epileptic encephalopathy (DEE) referred to as DEE78. Only eight patients have been reported globally. This study presents the clinical presentation and genetic analysis of a Chinese family with a child diagnosed with DEE78, due to a novel *GABRA2* variant.

**Methods:**

Genetic diagnosis was performed using trio‐whole exome sequencing, followed by bioinformatics predictions of pathogenicity. Structural modeling assessed the potential impact of the variant. A mutant plasmid was constructed and transfected into 293 T cells. Western blotting (WB) was used to evaluate mutant protein expression, while co‐immunoprecipitation (Co‐IP) analyzed interactions with GABRB3 and GABRG2 proteins. Immunofluorescence (IF) assessed the subcellular localization of the mutant protein.

**Results:**

The 6‐year‐old male proband presented with seizures starting at age two, along with global developmental delay and hypotonia. Genetic testing revealed a heterozygous de novo variant in *GABRA2* gene (NM_000807: c.923C>T, p.Ala308Val). Structural modeling suggested that this variant is located within the extracellular domain, which may disrupt hydrogen bonding interactions with GABRB3 and GABRG2. WB and Co‐IP showed reduced protein expression and impaired interactions, potentially destabilizing the pentamer receptor complex. If analysis revealed that the variant did not affect subcellular localization.

**Conclusion:**

This study identified a novel likely pathogenic *GABRA2* extracellular domain variant in a Chinese family causing the DEE phenotype. The results expand the genotypic and phenotypic spectrum of *GABRA2*‐related DEE.

## Introduction

Epileptic encephalopathies that present during infancy and childhood can manifest as seizures, development delays, intellectual disabilities, and various other neurological deficits. These conditions are categorized under the collective term of developmental and epileptic encephalopathies (DEE) to emphasize the intricate interplay between epileptogenesis and neurodevelopmental impairment.^1^, ^2^ Notably, even with effective seizure management, the neurological manifestations resulting from epileptic discharges may not completely resolve, and can, in fact, deteriorate progressively.^3^, ^4^, ^5^ DEE is significantly influenced by genetic factors, with defects in ion channels and receptors serving as primary etiological contributors. Such genetic variants disrupt the excitatory‐inhibitory balance within the brain by either enhancing excitatory activity or diminishing inhibitory control, thereby initiating and propagating seizures.^6^, ^7^ Gamma‐aminobutyric acid A receptors (GABA_A_Rs) are ligand‐gated chloride ion channels composed of pentameric assemblies consisting of 19 subunits (α1‐6, β1‐3, γ1‐3, ρ1‐3, δ, ε, π, θ) and primarily facilitate GABAergic inhibitory pathways in the central nervous system.^8^ GABA_A_Rs are critical targets for pharmacological interventions aimed at treating epilepsy, insomnia, anxiety, and panic disorders. These receptors are modulated by various agents, including benzodiazepines, barbiturates, anesthetics, neurosteroids, and ethanol. The predominant isoform typically comprises of two α1, two β2, and one γ2 subunit, with GABA binding at the β‐α interface and benzodiazepines binding at the α‐γ interface.^9^ Theoretically, the cortex c is capable of generating approximately 62,847 unique GABA_A_R subtypes through differential assembly, which influences the binding characteristics and functional outcomes of synthetic modulators. This receptor diversity arises from variations in subunit composition and interface affinities, which may be modulated by assembly factors or chaperones. Such diversity facilitates rapid adaptation to specific signaling demands, enhancing input recognition and the precise regulation of output. Distinct receptor subtypes can emerge across various anatomical locations, developmental stages, and physiological conditions, thereby contributing to the formation of complex neuronal circuits and behaviors.^10^ Pathogenic variants affecting different GABA_A_R subunits have been associated with a range of DEE syndromes. Known epilepsy‐associated variants have been identified in subunits α1‐3, α5 (*GABRA1, GABRA2, GABRA3, GABRA5*), β1‐3 (*GABRB1, GABRB2, GABRB3*), γ2 (*GABRG2*), and δ (*GABRD*).^11^, ^12^, ^13^ Among these, variants in *GABRG2* are the most prevalent, occurring in various syndromes, including Dravet syndrome, generalized epilepsy with febrile seizures plus, childhood absence epilepsy, and febrile seizures.^14^ In contrast, variants in *GABRA2* are exceedingly rare, with only seven distinct variants reported across seven unrelated families to date. The eight affected individuals carrying these variants predominantly exhibit DEE phenotypes.^13^, ^15^, ^16^, ^17^

GABAergic interneurons that target the soma and axon initial segment (AIS) are play a crucial role in the regulation of action potential generation. These interneurons release GABA, which binds to GABA_A_Rs, specifically α1‐containing GABA_A_Rs located on dendrites and the soma, and α2‐containing receptors found on the soma and AIS. Parvalbumin (PV)‐positive chandelier cells specifically target the AIS with α2‐containing GABA_A_Rs, engaging in interactions with collybistin and *FGF13* during the process of synaptogenesis. The *GABRA2*‐1 mutation observed in murine models disrupts the clustering of α2 receptors, resulting in developmental seizures, increased mortality, and anxiety‐like behaviors. Furthermore, disruptions in PV cell signaling have been associated with various neurodevelopmental disorders, including schizophrenia, autism spectrum disorders, Angelman syndrome, and Rett syndrome, all of which exhibit a high incidence of epilepsy.^18^ The α2 subunit of the GABA_A_R encoded by the *GABRA2* gene, forms Cys‐loop ligand‐gated ion channels characterized by a single polypeptide chain that spans four transmembrane domains (M1 to M4), an intracellular region, and a ligand‐binding extracellular domain (ECD).^19^ Importantly, all known variants of *GABRA2* are localized to the transmembrane region, indicating a potential hotspot region for pathogenicity. In this study, we present a genetic analysis of a family with DEE caused by a novel variant in the ECD of the *GABRA2* protein. To our knowledge, this represents the first investigation of the *GABRA2* variant within the Chinese population, thereby expanding the genetic spectrum and offering new insights into the genotype–phenotype associated with this rare disease.

## Materials and Methods

### Ethical considerations

All research involving human participants complies with the principles outlined in the 1964 Helsinki Declaration and its subsequent revisions, as well as other comparable ethical standards. This study has received approval from the Medical Ethics Committee of the First Affiliated Hospital of Anhui Medical University (Approval number: PJ2024‐04‐56). Informed consent has been obtained from patients and their families, who have also consented to the disclosure of their genetic variations and clinical data.

### Sample collection

Peripheral venous blood samples, each measuring 3 mL, were collected from the proband and his parents. Genomic DNA was extracted from these blood samples utilizing a DNA extraction kit (AU1802, Bioteke Corporation, Beijing, China) in accordance with the manufacturer's instructions. The concentration and purity of the extracted DNA were assessed using a Qubit 2.0 fluorimeter (Thermo Fisher Scientific, USA). Subsequently, the extracted DNA samples were then stored at −20°C until further analysis.

### Whole exome sequencing

Whole exome sequencing (WES) was conducted on the DNA samples utilizing the Illumina NovaSeq 6000 system (Illumina, USA). Exome capture was performed in accordance with the manufacturer's guidelines using the xGen Exome Research Panel v2.0 (IDT, Iowa, USA). The raw sequencing reads were aligned to the human reference genome (GRCh37/hg19) employing the Burrows‐Wheeler Aligner (BWA v0.7.10). Variant calling and annotation were executed using the GATK v3.5, Picard v1.128, and ANNOVAR tools. A filtering process was implemented to exclude common variants (allele frequency >1%) identified in public databases, including 1000 Genomes, ExAC, ESP, dbSNP, and gnomAD. The pathogenicity prediction of the remaining variants was carried out using MutationTaster, SIFT, Provean, M‐Cap, Revel, and PolyPhen‐2 software. A pathogenicity assessment of the variants was performed based on their category, population carrier frequency, and the guidelines established by the American College of Medical Genetics and Genomics (ACMG) guidelines for variant interpretation.^20^ Candidate pathogenic variants were subsequently validated through Sanger sequencing in both the proband and their parents.

### Sanger sequencing

Specific primers for the identified variants were designed utilizing Primer Premier v5.0 software. For the purpose of PCR amplification, the DNA fragment encompassing the variant site was targeted using the designed forward primer (5′‐*ATCCCAAGCCCATCCTCTTTTGGT*‐3′) and reverse primer (5′‐*AGTAAAAGCTTTGGGGGCTGGAGG*‐3′). The purified PCR products were subsequently subjected to bidirectional sequencing employing the BigDye Terminator v3.1 Cycle Sequencing Kit (Thermo Fisher Scientific) on the ABI 3730XL DNA sequencer (ABI, USA). The resulting sequencing data were then analyzed using Chromas Lite v2.01 software.

### Analysis of molecular dynamics simulations

Homology modeling was conducted utilizing the cryo‐electron microscopy (cryo‐EM) structure of the human GABA_A_R α1β3γ2 complex (PDB ID: 6HUP).^21^ The protein sequences for *GABRA2* (α2 subunit), GABRB3 (β3 subunit), and GABRG2 (γ2 subunit) were sourced from UniProt and aligned with the α1β3γ2 template sequence through the SWISS‐MODEL platform.^17^ The model of the α2β3γ2 complex was generated using Alphafold (https://alphafold.ebi.ac.uk/), and the identified variants were incorporated based on the wild‐type structure. Subsequently, molecular dynamics simulations were performed using GROMACS version 5.14, employing the GROMOS 53A6 force field. The simulations were conducted with the SPC water model over a total duration of 50 ns. Structural data structure figures were produced using PyMOL v2.5 and Origin v8.5. The gmx rms tool was utilized to calculate the root mean square deviation (RMSD) between the wild‐type and variant protein complexes, and the structural representations were visualized using PyMOL v2.5.

### Construction of plasmids

To construct the wild‐type GABRA2 plasmid (GABRA2‐WT), we employed the pECMV‐FLAG‐N vector and amplified the GABRA2‐WT gene utilizing Phanta® Max Super‐Fidelity DNA Polymerase (Vazyme #P505, China Novagen Company). The primers used for amplification included the upstream primer 5′‐aaggatgacgatgacaagcttATGAAGACAAAATTGAACATCTACAACA‐3′ and the downstream primer 5′‐ggtttaaacgggccctctagaTCAAGGACTGACCCCTAATACAGG‐3′. Subsequently, the GABRA2 mutant plasmid (GABRA2‐MUT) was constructed in accordance with the protocol outlined in the Mut Express MultiS Fast Mutagenesis Kit V2 (Vazyme #P505). The primers used for this amplification consisted of the upstream primer 5′‐CCCAAAGTGGtTTATGCAACTGCCATGGACTGG‐3′ and the downstream primer 5′‐ggtttaaacgggccctctagaTCAAGGACTGACCCCTAATACAGG‐3′. Following sequencing validation, the plasmids were amplified and extracted. Human 293T cells, obtained from the Shanghai Cell Bank, were cultured in high‐glucose DMEM supplemented with 10% fetal bovine serum (Gibco, USA). Once the cell density reached 70% in a 6‐well plate, plasmid transfection was conducted using Lipofectamine 3000 reagent (Thermo Fisher Scientific, MA, USA).

### Immunoblotting

Total cellular proteins were extracted from the transfected cells utilizing Radio‐Immunoprecipitation Assay (RIPA) lysis buffer (Thermo Fisher Scientific, MA, USA). The protein concentration was quantified using the BCA assay (Thermo Fisher Scientific). Subsequently, the proteins were then separated via 10% sodium dodecyl sulfate‐polyacrylamide gel electrophoresis (SDS‐PAGE) and transferred to a polyvinylidene fluoride (PVDF) membrane (Bio‐Rad Laboratories, CA, USA). Following a blocking step, the membrane was incubated overnight at 4°C with primary antibodies: DYKDDDDK Tag (9A3) Mouse monoclonal antibody (Cat#8146, Cell Signaling Technology, MA, USA, diluted 1:1000) and GAPDH (D4C6R) Mouse monoclonal antibody. The membrane was then incubated with an anti‐mouse IgG HRP‐linked secondary antibody (Cat#7076, Cell Signaling Technology, MA, USA, diluted 1:5000) at 25°C for 2 h. Finally, target bands were detected using a chemiluminescence substrate (Perkin Elmer, USA), and exposure and imaging were conducted using the Tanon‐5200 Multi‐system (Tanon, Shanghai, China).

### Immunofluorescence

Forty‐eight hours post‐transfection, the 293 T cells were transferred to confocal microplates (#BS‐20‐GJM, Biosharp, Hefei, China). The cells were subsequently fixed using a 4% paraformaldehyde tissue fixative (#P0099, Beyotime, Shanghai, China) for a duration of 15 min and permeabilized with 0.5% Triton X‐100 (#P0096, Beyotime, Shanghai, China) at 25°C for an additional 15 min. Following this procedure, the cells were incubated with primary antibodies at a dilution of 1:200 at 4°C for 12 h. Subsequently, the cells were treated with fluorescent secondary antibodies at a dilution of 1:200 at 37°C for 1 h. An appropriate volume of 4′,6‐diamidino‐2‐phenylindole (DAPI) staining solution (#C1005, Beyotime, Shanghai, China) was then applied for 5 min in the dark. Images were acquired using a confocal microscope (Leica TCS SP8, Germany). The primary antibodies utilized included the DYKDDDDK Tag (9A3) Mouse mAb (Cat#8146, Cell Signaling Technology, MA, USA) and the Calnexin Polyclonal antibody (Cat#10427‐2‐AP, Proteintech, Wuhan, China). The fluorescent secondary antibody employed was CoraLite488‐conjugated Goat Anti‐Mouse IgG (H + L) (Cat#SA00013‐4, Proteintech, Wuhan, China).

### Co‐immunoprecipitation

Forty‐eight hours post‐transfection of the 293 T cells, the cells were treated with RIPA lysis buffer and subsequently centrifuged at 12,000 g for 10 min at 4°C to facilitate the extraction of cellular proteins. The concentration of the protein lysates was determined using the Bradford assay (Bio‐Rad). A portion of the supernatant (300 μL) was retained as Input, while the remaining supernatant was incubated with 20 μL of Anti‐FLAG immunomagnetic beads (Cat#B26101, Selleck, TX, USA) for a duration of 2 h. The beads were then washed twice with Tris‐Buffered Saline (50 mM Tris HCl, 150 mM NaCl, pH 7.4). Following the addition of 50 μL of 1× loading buffer and subsequent boiling, Western blot (WB) analysis was conducted. The primary antibodies utilized included: DYKDDDDK Tag (9A3) Mouse mAb (Cat#8146, Cell Signaling Technology, MA, USA, diluted 1:200); GABRB3 Antibody (Cat#PB0627, Boster Biological Technology, Wuhan, China, diluted 1:1000); GABRG2 Antibody (Cat#A02361‐1, Boster Biological Technology, Wuhan, China, diluted 1:1000); and β‐Actin (8H10D10) Mouse mAb (Cat#3700, Cell Signaling Technology, MA, USA, diluted 1:1000). The secondary antibodies employed were: Anti‐mouse IgG, HRP‐linked (Cat#7076, Cell Signaling Technology, MA, USA, diluted 1:5000) and Anti‐rabbit IgG, HRP‐linked (Cat#7074, Cell Signaling Technology, MA, USA, diluted 1:5000).

### Statistical analysis

GraphPad Prism v8.0 software was utilized for statistical analysis. Comparisons between the two groups were performed employing Student's t‐test, with a significance threshold set at *P* < 0.05.

## Results

### Clinical presentation

**Figure 1:**
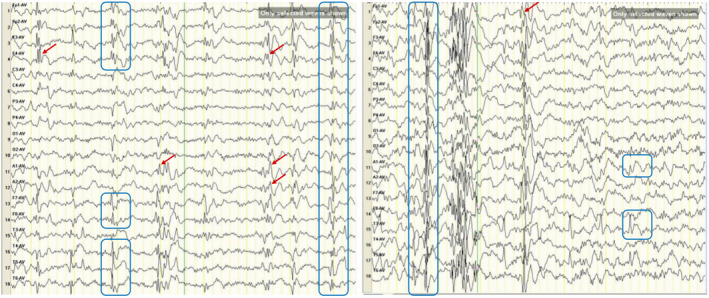
Interictal EEG findings. Slow background activity with generalized multifocal spikes (blue box), multi‐spikes (red arrow), and epileptoid waves.

The subject of this case study is a 6‐year‐old male patient, who is the second child of healthy, non‐consanguineous parents, and has no known family history of epilepsy. The patient was born at full term and subsequently developed jaundice. At 6 months of age, he exhibited unstable head control, an inability to sit independently, and hypotonia of the limbs. The onset of epileptic seizures occurred around the age of 2, initially characterized by episodes of eye staring, bilateral fist clenching, limb stiffening, and unresponsiveness, each lasting approximately 1 min before resolving spontaneously, followed by a return to normal mentation. Currently, the seizures manifest as episodes of blank staring, limb stiffening, and minimal responsiveness when his name is called, with a duration exceeding 10 s before resolution. At over 4 years of age, the patient was hospitalized due to recurrent convulsive seizures that had persisted for 2 years. A physical examination upon admission revealed no facial dysmorphism or neurological signs, and limb muscle strength and tone were grossly normal. Developmental milestones were notably delayed in comparison to peers, with unsteady gait, inability to call mom/dad, and only occasional vowel sounds for communication. Laboratory tests indicated a decreased red blood cell count and hemoglobin levels. Imaging studies, including chest and knee X‐rays, cardiac ultrasound, and electrocardiogram, did not reveal any significant abnormalities. Brain magnetic resonance imaging (MRI) was also unremarkable. Video electroencephalography (EEG) demonstrated a slow background activity with generalized multifocal spikes, multi‐spikes, and fast waves, which was accentuated during sleep (Figure 1). Inpatient treatment included the administration of vitamin B and neurotrophic drugs; however, the seizures persisted. Following discharge, the patient continued on antiepileptic medications, specifically sodium valproate and lamotrigine. At 6 years of age, the patient exhibits no speech, poor comprehension, an inability to run or jump, has difficulty squatting, and experiences small seizures when agitated, accompanied by frequent crying spells and irritability.

### Results of genetic testing

**Figure 2:**
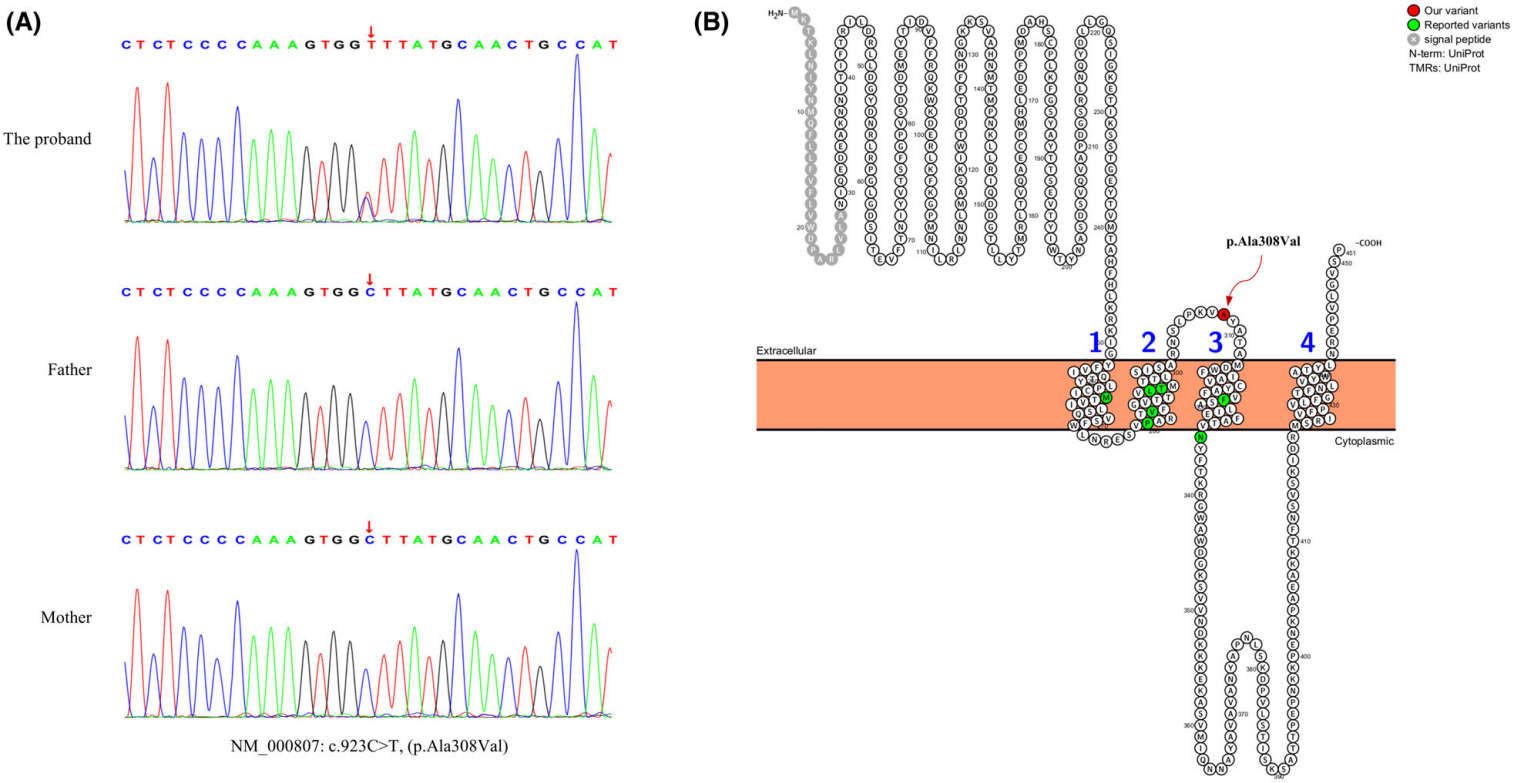
Molecular findings in the epilepsy family. (A) Sanger sequencing confirmed a heterozygous variation in the *GABRA2* gene (NM_000807: c.923C>T, p.Ala308Val) in the proband, while both parents exhibit the wild‐type allele. (B) The *GABRA2* variant Ala308Val is positioned in the extracellular region between M2 and M3 (indicated by the red arrow), whereas previously reported variants are primarily located in the transmembrane region (indicated by green circles).

WES revealed a heterozygous missense variant, c.923C>T (p.Ala308Val), located in the exon 9 of the *GABRA2* gene (NM_000807) in the patient. This variant was confirmed to be *de novo* through Sanger sequencing, as it was absent in both healthy parents (Figure 2A). Notably, this variant was not detected in various population databases, including ExAC, 1000 Genomes, dbSNP, or gnomAD. Furthermore, the gnomAD database indicates that the *GABRA2* gene has a missense_Z score of ≥ 3.09. In silico prediction tools have assessed the variant as deleterious, yielding disease‐causing scores of 1 from MutationTaster, 0.004 (damaging) from SIFT, 0.129 (damaging) from M‐CAP, 0.998 (probably damaging) from PolyPhen‐2 HDIV, 0.984 (probably damaging) from PolyPhen‐2 HVAR, 0.851 (deleterious) from REVEL, 28.8 (deleterious) from CADD, and −3.18 (deleterious) from Provean. Additionally, this variant is located in proximity to previously reported *GABRA2* gene variants (Figure 2B). According to the ACMG guidelines, this variant is classified as likely pathogenic based on the following criteria: PS2_moderate, PM1, PM2_supporting, PP2, and PP3. Protein analysis and visualization conducted using the Protter database (https://wlab.ethz.ch/protter/start/)^22^ revealed that the Ala308Val variant is situated in the extracellular region between transmembrane segments M2 and M3. This variant has not been previously reported, thus categorizing it as a novel variant (Figure 2B).

### Results of structural simulation analysis

**Figure 3:**
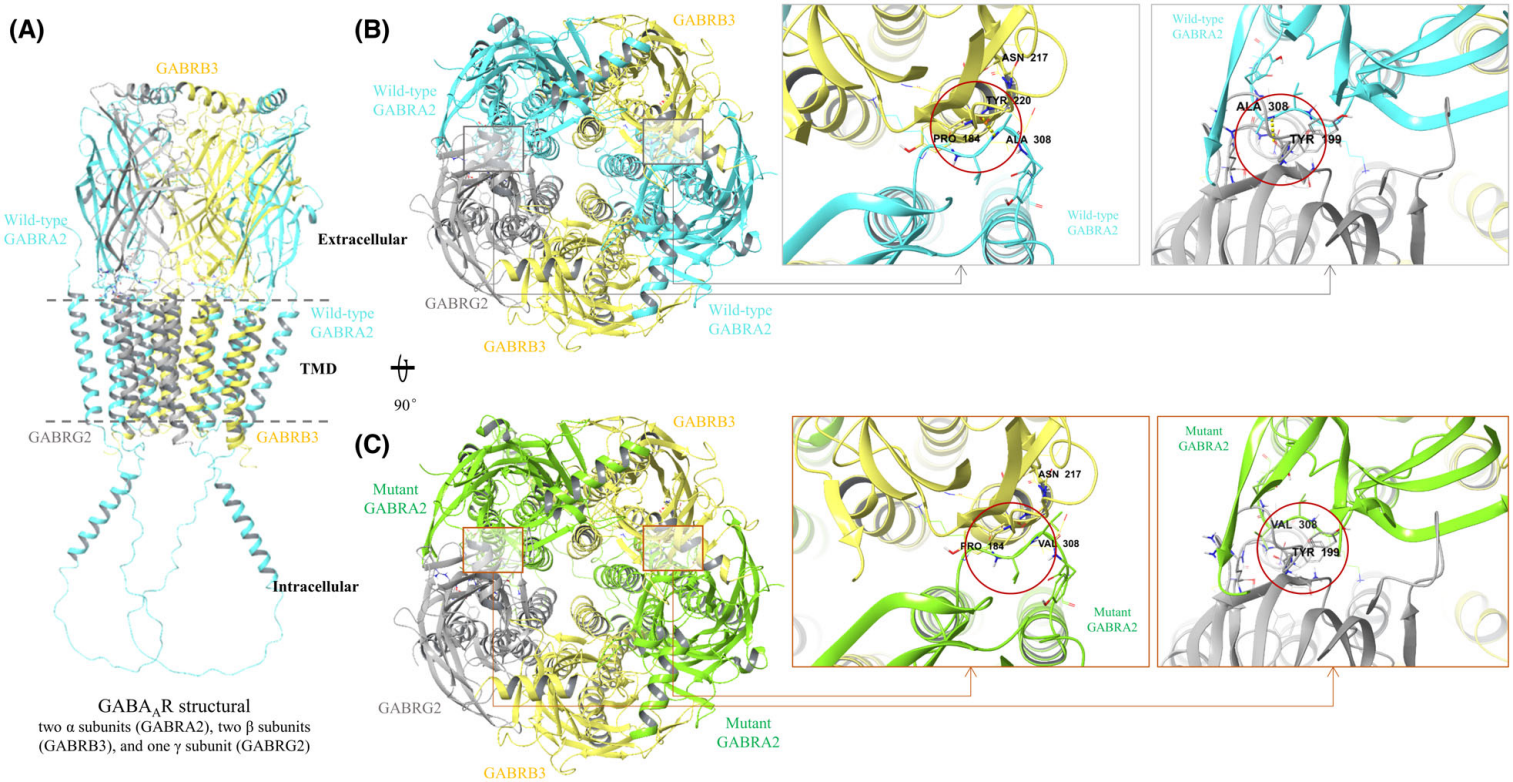
Analysis of GABA_A_R pentameric structure. (A) A pentameric model featuring 2 α2 subunits (GABRA2), 2 β3 subunits (GABRB3), and 1 γ2 subunit (GABRG2). (B) The structure is rotated 90 degrees for a top‐down view. In the wild‐type GABRA2, Ala308 forms hydrogen bonds with Pro209 of GABRB3 and with another GABRA2 protein at position 238 of GABRG2. (C) In the mutated GABRA2 (p.Ala308Val), the longer valine side chain disrupts these hydrogen bonds, potentially altering structural stability.

As there is currently no available structure representation for α2‐containing GABA_A_Rs, we employed the Cryo‐EM structure of the human α1β3γ2 GABA_A_R to develop a model of an α2‐containing pentameric transmembrane chloride channel. This model comprised two α2 subunits, two β3 subunits, and one γ2 subunit. Although the predominant isoform in the adult brain is the α1β2γ2 GABA_A_R and α2‐containing GABA_A_Rs, while less abundant, are present and may fulfill distinct physiological roles. Molecular dynamics simulations were conducted to evaluate the structural implications of the p.Ala308Val variant identified in the *GABRA2* gene. The *GABRA2* protein variant Ala308Val is situated in the extracellular region. The protein structures achieved equilibrium after 50 ns with an average root mean square deviation (RMSD) of 4.5 Å observed between the wild‐type and mutant complexes. In the wild‐type GABA_A_R structure, within the interaction interface between *GABRA2* and GABRB3 proteins, Ala308 of *GABRA2* forms stabilizing hydrogen bonds with Pro209 [auth 184] of GABRB3, as well as with Tyr238 [auth 199] of GABRG2. The introduction of the bulkier valine side chain at position 308, through in silico mutagenesis, results in steric clashes that disrupt both these hydrogen bonds. This alteration is predicted to destabilize the interface between the α2 and β3 subunits, as well as between the α2 and γ2 subunits. Given that inter‐subunit interactions are critical for maintaining the integrity of the pentameric receptor complex, the p.Ala308Val variant may compromise the proper assembly and structural stability of the GABA_A_R (Figure 3). Our simulation analysis elucidated the molecular mechanism by which this variant may impair GABA_A_R function, potentially leading to reduced inhibitory neurotransmission and neuronal hyperexcitability, which underlie the epilepsy phenotype observed in the patient.

### The *GABRA2* variant may not have a significant impact on the function of intracellular localization

**Figure 4:**
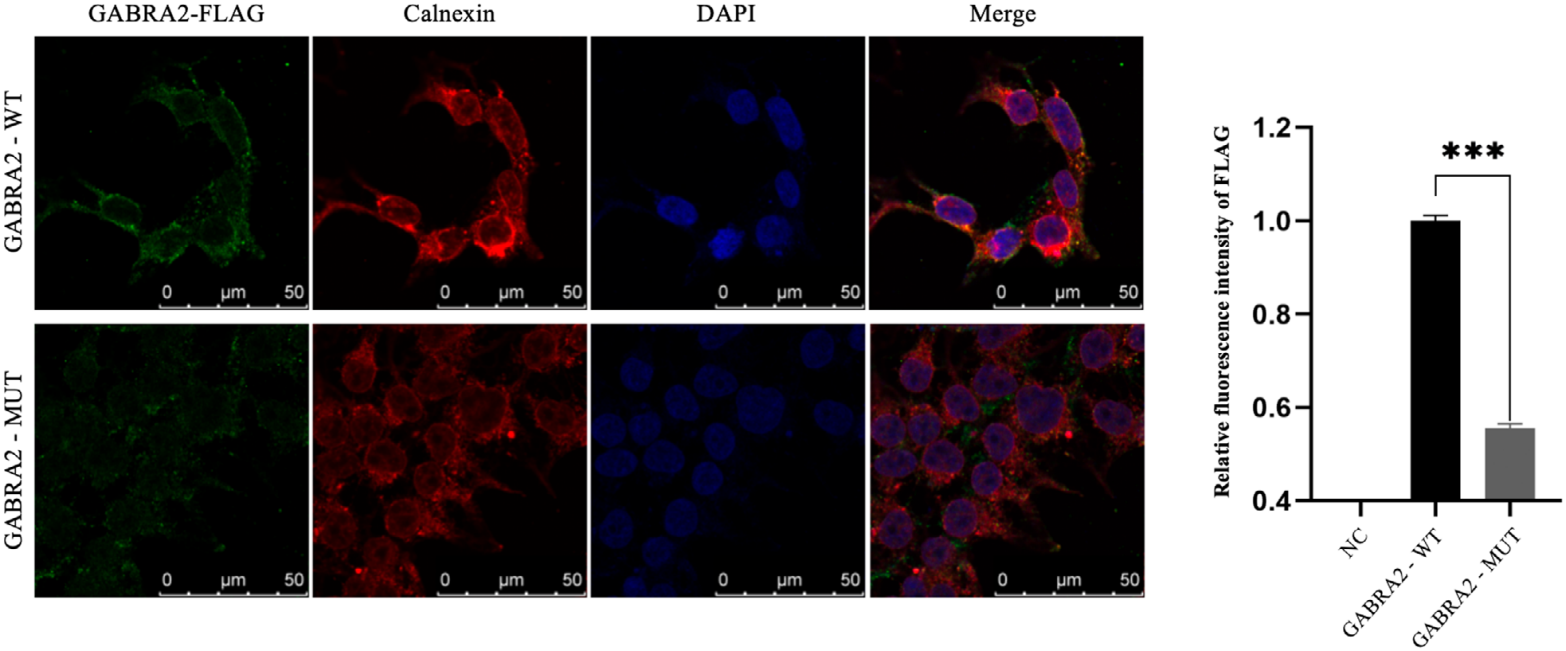
Subcellular localization of the GABRA2 Variant in 293T Cells. GABRA2 with FLAG‐tag (green), calnexin antibody (red, ER marker), and DAPI (blue, nuclear counterstaining). Confocal microscopy indicated that both wild‐type and variant proteins localized to ER vesicles, but the variant showed reduced expression and weaker fluorescence. Normalized relative fluorescence intensity analysis revealed significant differences between GABRA2‐WT and GABRA2‐MUT groups, ***P < 0.001.

To assess the intracellular localization of the GABRA2 variant, we conducted an analysis of transiently transfected wild‐type and mutant cells utilizing immunofluorescence techniques, co‐staining with calnexin, a marker for the mature endoplasmic reticulum (ER). The findings indicated that both GABRA2‐WT and GABRA2‐MUT were predominantly localized in proximity to ER vesicles or within the ER, implying that the GABRA2‐MUT does not significantly alter ER localization. Nevertheless, a notable reduction in expression levels was observed, with the fluorescence intensity of GABRA2‐MUT being approximately 44.4% lower than that of GABRA2‐WT (Figure 4).

### The *GABRA2* variant may influence the interaction with GABRB3/GABRG2 proteins

**Figure 5:**
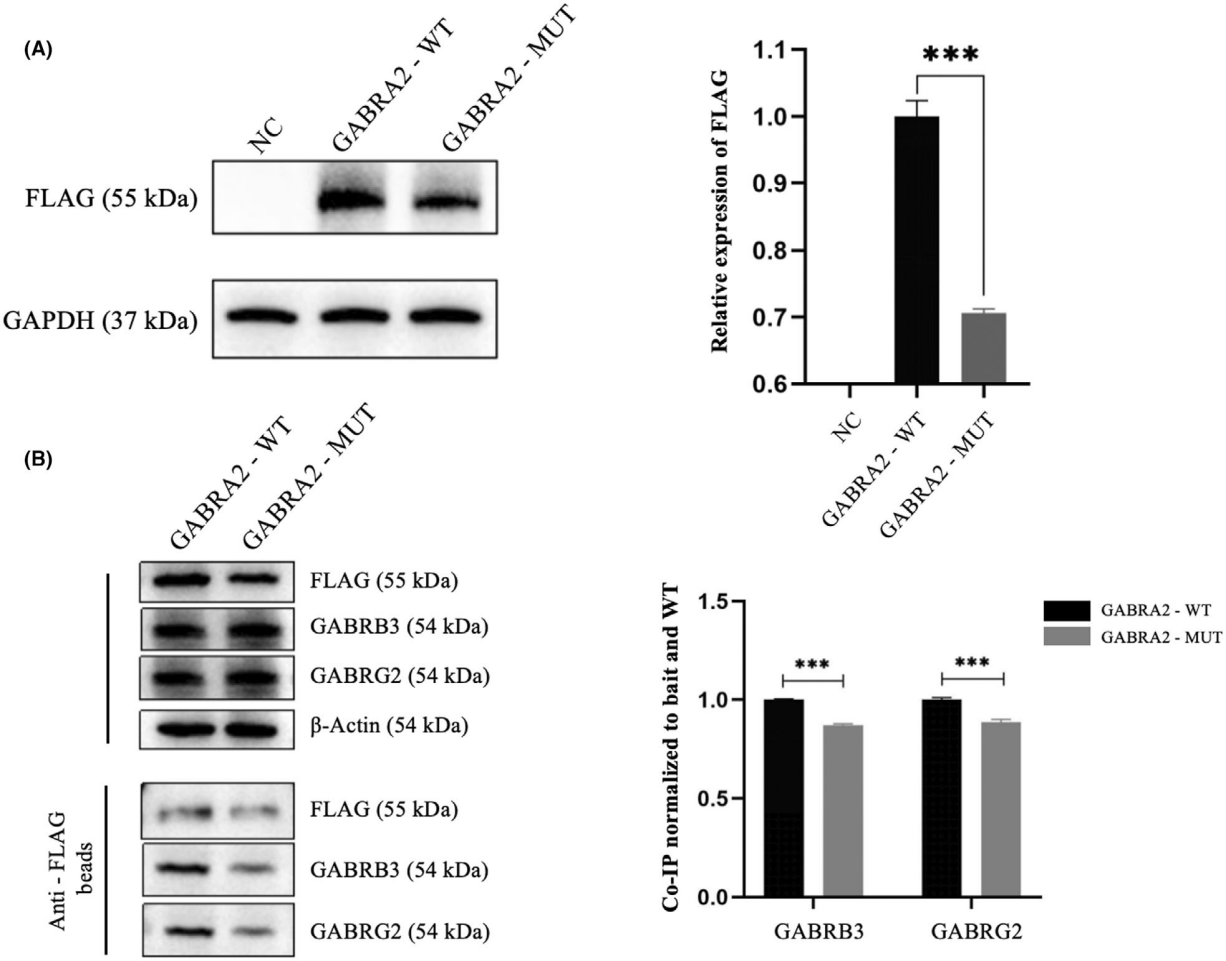
In vitro functional assay. (A) Western blotting analysis indicated that the GABRA2‐MUT group exhibited protein expression, but it was significantly lower than the GABRA2‐WT group, with a reduction of approximately 70.62% (***P < 0.001). (B) CO‐IP results showed that wild‐type GABRA2 interacted with GABRB3/GABRG2, while the mutant may disrupt this interaction. The right panel presents normalized CO‐IP analysis, ***P < 0.001.

To assess the impact of the p.Ala308Val variant on GABRA2 protein expression, we performed Western blot analysis on 293 T cells that were transfected with either wild‐type (GABRA2‐WT) or mutant (GABRA2‐MUT) expression constructs. The immunoblotting results confirmed protein expression for both variants; however, the GABRA2‐MUT group exhibited a significant reduction in expression, approximately 70.62% lower than that of the GABRA2‐WT group (Figure 5A).

To examine the impact of the variant on the GABA_A_R complex, we conducted an analysis of the interaction between GABRA2‐MUT and the GABRB3/GABRG2 subunits through co‐immunoprecipitation. The results obtained from Western blot indicated that GABRA2‐WT exhibited a robust interaction with the GABRB3 and GABRG2 proteins. In contrast, the GABRA2‐MUT markedly impaired this interaction (Figure 5B).

## Discussion

We present a case involving a Chinese male child who exhibited epileptic seizures and global developmental delay. Trio‐based WES identified a heterozygous de novo variant (p.Ala308Val) in the *GABRA2* gene, which has been linked to the rare disorder known as DEE78 (OMIM#618557). Functional experiments conducted in vitro indicated that this variant may result in reduced expression levels and could potentially disrupt the interaction with GABRB3/GABRG2, which could potentially disrupt the structural stability of the GABA_A_Rs. Only eight patients with *DEE78* have been documented so far (Supplemental Table S1). The primary phenotype associated with this condition includes primary phenotype associated with this condition includes the onset of seizures and hypotonia within the first 6 months of life, accompanied by developmental delay and intellectual disability that tends to deteriorate over time. Additionally, some patients may present with intractable seizures and characteristics of autism spectrum disorder.^13^, ^15^, ^16^, ^17^ In the case presented, the patient experienced his initial seizure after the age of two and exhibited severe global developmental delay, which aligns with the phenotypic manifestations of DEE78. Although his seizures were effectively managed with sodium valproate and lamotrigine, his cognitive deficits persisted and remained unchanged.

The *GABRA2* gene is situated on chromosome 4p12 and encodes the α2 subunit of the γ‐aminobutyric acid A receptor. This α2 subunit consists of 451 amino acids and exhibits high expression levels in the brain, particularly within the prefrontal cortex, where it plays a crucial role in inhibitory neurotransmission (https://www.proteomicsdb.org/proteomicsdb/#human/proteinDetails/P47869/expression).^23^ The α2 subunit combines with other subunits (β, γ) to form pentameric chloride channels that facilitate inhibition in the central nervous system.^24^ Structurally, the α2 subunit features an extracellular N‐terminal ligand‐binding domain, four transmembrane domains (M1‐M4), and an intracellular loop situated between M3 and M4 (ICD).^16^ The novel heterozygous p.Ala308Val variant identified in our patient is localized to the extracellular region between the transmembrane helices M2 and M3. A high‐resolution cryo‐EM structure of a predominant synaptic isoform, α1β3γ2, provided the foundation for a structural analysis of this variant. By aligning with the α2 structure, a model of the α2β3γ2 complex was constructed. Structural analysis suggests that this substitution may disrupt the inter‐subunit interactions that stabilize the pentameric receptor, potentially altering its structural stability and affecting its biological function. A recent study examined variants in the *GABRA1* gene utilizing the sa43718 allele in zebrafish. This nonsense variant resulted in decreased expression of gabra1 and other α subunits, including *GABRA2*; however, larvae still exhibited responses to pentylenetetrazole, indicating residual GABA_A_R activity. Proteomics analyses revealed abnormal protein expression, corroborating the involvement of GABA_A_R α subunits in seizure‐like phenotypes associated with dysfunctional inhibitory synapses.^25^ Another investigation focused on impaired GABAergic regulation in interneurons derived from the medial ganglionic eminence in the context of the tuberous sclerosis complex. Specifically, somatostatin‐expressing interneurons demonstrated upregulation of the *GABRA2*, which may lead to reduced receptor affinity and potentially impact inhibitory neurotransmission within this specific subpopulation.^26^ Simulation analyses conducted in this study indicated that the p.Ala308Val variant disrupts hydrogen bonds at both the β‐α interface for GABA binding and the α‐γ interface for benzodiazepine binding, thereby providing insights into the molecular mechanisms underlying GAB_A_ARs dysfunction in epilepsy. Nevertheless, the understanding of α2‐containing GABA_A_Rs remains limited, highlighting the need for future mechanistic investigations into GABAergic signaling and pharmacology.

The GABAA receptor is a ligand‐gated chloride ion channel that belongs to the pentameric Cys‐loop ion channel superfamily.^27^ This receptor is composed of five subunits selected from a total of 19 isoforms, which collectively form a central ion channel.^28^ The proper functioning of GABA_A_R is contingent upon the accurate synthesis, assembly, and membrane targeting of its subunits, all of which are critical for GABAergic inhibitory signaling.^29^ The α2 subunit represents one of six isoforms that can occupy the principal subunit position.^30^ Although the precise role of the α2 subunit remains incompletely understood, the M3‐M4 intracellular domain (ICD) appears to facilitate receptor clustering at synapses via interactions with scaffolding proteins like collybistin and gephyrin.^31^ Only seven pathogenic variants have been identified in *GABRA2* to date, all of which are located within the transmembrane region.^13^, ^15^, ^16^, ^17^ Variants situated within the transmembrane segments (TM) may influence ion channel function by altering the structural integrity and current amplitude of the channel. For instance, the p.Pro280Leu variant, located in the M2 helices near the channel pore, has been shown to reduce the aperture of the desensitization gate, thereby affecting ion channel functionality.^17^ Other transmembrane variants, including p.Met263Thr, p.Val284Ala, p.Leu291Val, p.Thr292Lys, and p.Phe325Leu, have been associated with diminished GABA‐evoked current amplitudes, suggesting a loss of protein function.^13^, ^16^ The composition of synaptic and extrasynaptic GABA_A_Rs varies, which in turn influences their inhibitory functions. For instance, synaptic GABA_A_Rs, characterized by the presence of γ subunits, mediate phasic inhibition that accounts for approximately 25% of inhibitory conductance.^32^ The p.Ala308Val variant identified in our patient is predicted to disrupt inter‐subunit interactions, as indicated by structural simulation analyses. Experimental findings demonstrate reduced expression levels and impaired interactions with GABRB3 and GABRG2, although subcellular localization remains unaffected. The observed reductions in GABA_A_R expression may result in chloride imbalance and excitatory GABAergic signaling, which are implicated in various disorders, including neurodevelopmental, neurodegenerative, and psychiatric conditions.^33^, ^34^ In vitro findings are consistent with in vivo evidence from GABA_A_R α1, α3, or γ2 knockout mice, which exhibit significant structural alterations in inhibitory synapses. Furthermore, CRISPR‐Cas9‐mediated genetic deletion of GABA_A_Rs in a single hippocampal neuron leads to substantial reductions in GABAergic synapses, highlighting the essential role of GABA_A_Rs in synapse development.^35^ Additional electrophysiology studies are necessary to comprehensively define the functional impacts and to develop targeted treatment strategies for *GABRA2* variants. Understanding the modifications in receptor properties induced by different variants will enhance insights into the genotype–phenotype correlation in this complex yet rare disease. Moreover, further research is warranted to elucidate the changes in GABA_A_R subunits during seizure activity, as prolonged seizures can result in severe brain damage. During seizures, calcium influx through ionotropic glutamate receptors triggers the rapid internalization of synaptic GABA_A_Rs, thereby weakening inhibition and exacerbating seizure activity. GABAergic interneurons are particularly susceptible to excitotoxic death, which can lead to hyperexcitable neuronal circuits and disrupted brain rhythms, underscoring the importance of protecting these interneurons to prevent the onset of epilepsy.^36^

Among the various GABA_A_R subunits, pathogenic variants in the *GABRG2* gene are the most commonly observed. However, mutations in other subunits, such as *GABRA1, GABRB2*, and *GABRB3*, have also been frequently documented. In contrast, variants in *GABRA2* are an exceedingly rare cause of epilepsy, with only eight reported cases to date.^13^, ^15^, ^16^, ^17^ Initial genotype–phenotype analyses of GABA_A_R subunit defects have demonstrated a correlation between the localization of the variant and the severity of the disease. Patients with variants located in the transmembrane domain typically present with severe refractory epilepsy and profound intellectual disability, whereas those with variants in the extracellular domain tend to exhibit a milder phenotype. Notably, all known variants in *GABRA2* are situated within the transmembrane region and are associated with severe DEE characterized by drug‐resistant seizures. Our case represents the first documented extracellular variant in the *GABRA2* gene. Consistent with the genotype–phenotype patterns observed in other subunits, our patient displayed a relatively milder epilepsy profile, achieving good seizure control with the administration of valproic acid and lamotrigine. This suggests that the location of the variant may influence both disease severity and treatment response. Further investigations into the structure–function relationships of *GABRA2* mutations are essential for improving prognosis and facilitating personalized therapeutic strategies. Additionally, the reporting of further cases is crucial to establish robust genotype–phenotype correlations for this rare condition.

Currently, there is no specific treatment available for patients with variants in the *GABRA2* gene. The primary therapeutic strategy involves the use of antiepileptic drugs (AEDs) to achieve symptomatic control of seizures. This empirical approach is analogous to the management of other genetic epilepsies associated with dysfunction in GABA_A_Rs. However, a personalized precision medicine approach must take into account the specific functional consequences of the variant on inhibitory neurotransmission. Various AEDs, including topiramate, valproic acid, and clobazam, exert distinct effects on the GABAergic system, either by inhibiting reuptake, preventing degradation, or directly modulating GABA_A_Rs.^37^ Consequently, a comprehensive understanding of the channelopathy is essential for predicting treatment responses. As additional cases are documented, the potential for targeted therapies, such as gene replacement, may become feasible. It is crucial to elucidate the pathophysiology of rare variants in epilepsy‐related genes to advance precision medicine for affected individuals.

In conclusion, we have presented a case of developmental and epileptic encephalopathy in a male patient from China, who was found to possess a novel *de novo* heterozygous variant in the extracellular domain of *GABRA2*. This finding marks the first identification of a *GABRA2* variant located outside the transmembrane region, thereby contributing to the understanding of the allelic heterogeneity associated with this rare disorder. The identified substitution is predicted to disrupt the local protein structure and the inter‐subunit interactions that are essential for stabilizing the pentameric receptor complex. This case offers valuable insights into the structure–function relationships within the extracellular domain and contributes to the limited existing literature on the genotype–phenotype correlation for *GABRA2*‐associated epilepsy. The relatively milder phenotype observed suggests that the location of the variant may modulate its effects. Further investigations into the precise functional implications of this variant are crucial for facilitating personalized therapeutic strategies. The documentation of additional cases will enhance genetic diagnosis, counseling, and the fundamental understanding of this rare and debilitating condition. Elucidating the pathophysiological mechanisms underlying *GABRA2* dysfunction is a critical step toward developing targeted treatments aimed at improving outcomes for affected individuals.

## Author Contributions

L.Y.: Case collection, formal analysis, article framework, methodology, software, validation, and writing‐original draft. X.W., R.H., J.J., and B.W.: Formal analysis, data collection, investigation, validation, and writing‐original draft. R.T. and D.W.: Funding acquisition, investigation, methodology, project administration, resources, supervision, validation, and writing–review and editing. All authors granted final approval for the manuscript.

## Funding Information

This work was funded by the National Natural Science Foundation of China (General Program) (No. 81472167).

## Conflict of Interest

The authors have declared no conflicts of interest.

## Supporting information


**Table S1.** Clinical features and *GABRA2* variants in patients with developmental and epileptic encephalopathie (DEE) (*n* = 9).

## Data Availability

All data generated or analyzed during this study are included in this published article and its supplementary information files. The datasets used and/or analyzed during the current study are available from the corresponding author upon reasonable request. No restrictions apply to the data, and they can be used for academic purposes with appropriate citation of the original source.
