# Supplementary material for: A novel de novo GABRA2 gene missense variant causing developmental epileptic encephalopathy in a Chinese patient
Source: Ann Clin Transl Neurol. 2024 Dec 31;12(1):137–48. doi: 10.1002/acn3.52262 (PMC11752098; doi:10.1002/acn3.52262)
Supplement: Supplementary file 1 — Table S1. Clinical features and GABRA2 variants in patients with developmental and epileptic encephalopathie (DEE) (n = 9). [file ACN3-12-137-s001.zip › Supplementary Table.docx]

**Table 1**. Clinical features and *GABRA2* variants in patients with developmental and epileptic encephalopathie (DEE) (n = 9).

| P． | Sex | Age | CDS | Protein | Inheritance | Exon | Domain | Seizure onset age, types | EEG | DD/ID/ASD | Brain MRI | Anti-epilepsy treatment | References |
| --- | --- | --- | --- | --- | --- | --- | --- | --- | --- | --- | --- | --- | --- |
| 1 | M | 6 ys | c.923C>T | p.Ala308Val | *de novo* | 9 | Loop TM2-3 | 2 years: blank staring, limb stiffening, minimal response on calling his name, lasting 10+ seconds before resolving | Slow background with multifocal spikes, spike-and-wave complexes, and fast activity, accentuated in sleep | Severe ID, neonatal hypotonia, no speech, not able to run and jump, jaundice | Normal | Seizure free  (VPA, LTG) | Present study |
| 2 | M | 10 ms | c.1003A>C | p.Asn335His | *de novo* | 9 | Loop TM3-4 | 10 weeks: mastication and blinking; hypertonus, grimacing and facial flushing, partial tonic with clonic eyelid movements | Fast background activity and left centro-parietal spikes | Severe ID, severe hypotonia, neonatal hypothermia, choreiform movements, vision impairment | Normal | Partial response  (TPM,GVG) | Orenstein et al., 2018 |
| 3 | F | 11 ys | c.875C>A | p.Thr292Lys | *de novo* | 9 | TM2 | 6 weeks: clustered focal seizures, spasms; tonic, tonic-clonic and myodonic | Slow and disorganized background with multifocal epileptiform discharges | Profound ID, severe hypotonia, no speech, not able to walk, microcephaly, spasticity, cortical visual impairment | Hypomyelination | Drug-resistant  (VPA,PB,CLB) | Butler et al., 2018 |
| 4 | F | 5.5 ms | c.851T>C | p.Val284Ala | *de novo* | 8 | TM2 | 1 month: ocular revulsion; absences, clonic | Isolated spike, rapid rhythm | Severe ID, hypotonia, pyramidal syndrome, failure to thrive, respiratory distress | Normal | Drug-resistant  (VPA,PHT) | Maljevic et al., 2019 |
| 5 | F | 32 ms | c.871C>G | p.Leu291Val | *de novo* | 9 | TM2 | 1 day: ocular version, focal clonic seizures; behavioral arrests, clonic, tonic; status epilepticus | Multifocal spikes right and left hemisphere; generalized spikes and polyspikes | Profound ID, severe hypotonia, no speech, not able to walk, reduced reflexes, cortical visual impairment | Atrophy, myelination, thin corpus callosum | Drug-resistant  (PB, CLB, CLN, VPA,TPM) | Maljevic et al., 2019 |
| 6 | F | 5 ys | c.788T>C | p.Met263Thr | *de novo* | 8 | TM1 | 2 days: focal clonic; spasm, clonic; status epilepticus | Discontinous, diffuse spike | Severe ID, hypotonia, respiratory distress, little speech, not able to walk, ASD | Normal | Drug-resistant  (Sabril, hydrocortisone) | Maljevic et al., 2019 |
| 7 | M | 17 ys | c..975C>A | p.Phe325Leu | *Father (mosaic)* | 9 | TM3 | 17 years: generalized tonic-clonic | normal, bilateral frontal/ fronto-central sharp activity, but not truly epileptiform | Moderate ID, ASD, language deday, jaundice | Normal | Seizure free  (LTG) | Maljevic et al., 2019 |
| 8 | F | 13 ys | c..975C>A | p.Phe325Leu | *Father (mosaic)* | 9 | TM3 | 2 years: complex partial; status epilepticus | NA | Mild - moderate ID, slightly language delay | Normal | Seizure free  (OXC) | Maljevic et al., 2019 |
| 9 | F | 10 ys | c.839C>T | p.Pro280Leu | *de novo* | 8 | TM2 | 15 months: tonic with upward eye deviation, or eye and head deviation to either side | Slow and disorganized background without paroxysmal activity | Severe impariment of language, global hypotonia, ASD, hyperactivity, behavior disturbances with repetitive movements, congenital nystagmus | Normal | Seizure free  (VPA, CLB) | Sanchis-Juan et al., 2020 |

Abbreviations: TM, transmembrane; DD, developmental delay; ID, intellectual disability; ASD, autism spectrum disorder; VPA, valproate; LTG, lamotrigine; TPM, topiramate; GVG, gamma vinyl-GABA; PB, phenobarbital; CLB, clobazam; PHT, phenytoin; CLN, clonazepam; OXC, oxcarbazepine.
